# Medical and Physical Intelligence

**Published:** 1825-08

**Authors:** 


### MEDICAL AND PHYSICAL INTELLIGENCE.

### PATHOLOGY.

1. *Pulsation of the Superficial Veins.*—Dr. Beyer, of Ohlau, relates the case of a soldier, twenty-six years of age, of a robust constitution, who had served some years, and enjoyed good health, until the month of December, 1815, when he was taken with paralysis of the right side, attended with evident signs of congestion in the head, and a generally increased impetus of the circulation, with occasional pains in the chest, and some cough: the intellects were perfectly sound. These symptoms varied in intensity until the 23d of January, when an universal pulsation of the veins was remarked; the pulse at the time being full, hard, and difficult to compress, and beating eighty-six in the minute. The patient was perfectly sensible, the breathing free, the face red, tongue dry, &c. The veins presented pulsations that were isochronous with those of the arteries; the whole skin was raised and fell with each pulsation: even in the eyes and tongue it was visible. There were no palpitations of the heart, but its pulsations corresponded to those of the arteries and veins: these last did not appear to be more than usually full. At the end of five days this phenomenon disappeared. In the evening, the head seemed to be affected, and a comatose state came on. The venous pulsation returned on the 1st of February, but the patient continued completely insensible. On the 2d, the motion and sensibility of the paralysed limbs was restored; the venous pulsation ceased the next day, as did the motions of the limbs; but the coma persisted, and on the 4th the patient died in this condition, after coughing two or three times.

We are informed that active bleeding, nitre, mineral acids, calomel, digitalis, and some antispasmodic medicines, had been employed in the above case; but how, or to what extent, or whether with any mitigation of the symptoms, does not appear.

Dissection threw no light upon this case: all the parts were sound, excepting that the middle semilunar valve of the left ventricle of the heart was ossified.—(*Journal Comp.* Juin.)

### THERAPEUTICS.

2. *Employment of Caustic to destroy the Variolous Eruption.*—[redacted] to the Royal Academy of Medicine, tending to prove that, if the pus[redacted] the two first days of their appearance, they die away entirely; and, if this is done even later, their duration is abridged, and no traces of them are left. The caustic he employs is a solution of nitrate of silver, in which he dips a probe, with which he pierces the centre of each pustule. M. Dumeril says that he has been long familiar with this practice, but, instead of the solution, he employed the solid caustic itself.—(*Archives Generales*, Juin.)

3. *On the Use of [the Carob*; by POWELL CHARLES BLACKETT, Esq. Surgeon R.N. M.R.C.S. &c. &c.—As several ancient authors have treated on the carob, and as nothing (that I am acquainted with) has hitherto appeared on this subject among English authors, I am led to suppose that a candid statement of the nature and uses of it might be acceptable to the profession or the public.

The Carob, Caroba, or Sweet Pod, is the produce, according to Linnæus, of the *ceratonia siliqua*, the systematic name of the plant, which affords the ceratium, or fruit: it grows principally in Sicily and Naples, as well as on the various coasts of the Mediterranean. Various authors describe the carob:—Ceratonia, χερατονια, Galeno arbor est, quæ Latinis siliqua dicitur, cujus fructus etiam. Græco nomine Τὰ χερατα et χερατια appellatur, propterea quod corniculum similitudine referat.

Ceratium, χερατιον; ceration item Græcis appellatur abor siliquas ferens corniculum similitudine unde et nomen habet. Latini siliquam Græcam appellant. COL. lib. de Arb. c. 25, siliquam Græcam, quam quidem χερατιον vocant, deinde Persicam ante brumam per autumnum scrito. PLIN. lib. 18, de fab[illegible]is loquens ait. Nam siliquæ caulesque gratissime sunt pabulo pecori. Item siliquæ χερατιον, sive siliquæ Græca, fructus et arboris, quæ a Galeno χερατωνια appellatur; siliquas tectus falcatis humani digiti longitudine, usque prædulcibus, et ob id in cibis expetitus, quum semen propter amaritudinem negligatur. Vide PLIN. lib. 15, cap. 24; HORAT. ii. Epist. 1.

——Vivit siliquis et pane secundo.

*Ceratonia*, carob-tree, or St. John's bread.*

*Carob-bean*, a fruit, whose taste is somewhat like a sweet chestnut. —BAILEY.

*Carobo*, the carob-tree, or fruit.—OFF.

*Ceratonia*, GERARD; abor quæ [illegible] Ceratia. The carob-tree; caroba; bean-tree, or St. John's bread; the fruit whereof is called Ceratium. fert

The pod, when unripe, is green and fleshy; and, when ripe, is still green with a mixture of brown, and, instead of being fleshy, becomes firm in its texture. When dried, the pod is compressed and unequal; about five or six inches long, and generally bent; of a red brown colour externally. The pulp is soft, having the colour of honey, and taste; has about thirteen or fourteen cells, in each of which is lodged a single oval and obtuse seed, excessively hard, of a reddish brown colour, of a bitter and hydrocyanic taste. These stones or seeds† are termed *xylococca*.

The carob, when fresh or green, is aperient in its properties: a decoction of it has been advantageously used in clysters, and, taken internally, is a very gentle laxative. When dry, if the bark of the pod be swallowed after mastication, it impedes [illegible]digestion, and proves astringent. The pulp of this bean is found to be demulcent, emollient, pectoral, and slightly expectorant; highly useful in asthmatic com-

* Supposed to be the fruit St. John used for bread when he was shipwrecked.

† If the seeds are roasted, they form a very good substitute for coffee.

plaints and convulsive coughs, particularly to children in the pertussis, and may be given according to the following manner:—Take of carob, well bruised, one ounce; add to it one pint and a half of water; let it boil gently down to a pint, and strain; of which give the patient a wine-glassful every three or four hours; or, as it is palatable, it can be used for children as a common drink. It will by these means overcome the cough, in a very easy and pleasing way.

For asthmatical patients, it will be found of greater service if prescribed with the decoction of the Iceland moss.—Take of the carob, well bruised, two ounces; add to it two pints of the decoction of the lichen Islandicus; let it boil gently down to a pint, and strain: of which give the patient three or four table-spoonsful every four or six hours.

It formerly constituted one of the ingredients of the Syrupus diacodii; and would, in my opinion, at the present day, be of more efficacy than sugar in the formation of the syrup of white poppies.

---

### MISCELLANEOUS.

4. *Report of the Associated Apothecaries.*—At the annual general meeting of the Associated Apothecaries and Surgeon-Apothecaries of England and Wales, held, by public advertisement, at the Crown and Anchor Tavern, Strand, July 6th, 1825, JOSEPH HAYES, Esq. President, the General Committee presented the following Report:—

"In conformity with established usage, your committee has the honour to lay before the general meeting a report of its proceedings during the last year.

"Anxious to effectuate the important objects for which it was appointed, your committee has deliberated, at various times, on the best means of promoting the public welfare, and ameliorating the condition of the general practitioner, by improving his education and increasing his attainments.

"The imperfections and disadvantages of the Act of 1815, commonly called 'the Apothecaries' Act,' have been so often alluded to, that it is unnecessary, on this occasion, to enter into any detail of them.

"Since the last general meeting, a Bill has been brought into Parliament, at the instance of the Society of Apothecaries, to amend the Act of 1815; but your committee has to regret that, previously to its introduction into the lower House, no opportunity was offered your committee of proposing those ameliorations which the public good, and justice to the general practitioner, alike require: neither was any intimation given (unless a verbal communication, avowedly unauthorised, may be so termed,) of the objects to be obtained by the proposed alterations. Thus circumstanced, your committee felt it their duty to seek, through proper channels, precise information, and to endeavour to obtain, by representation to the legislature, those alterations which should conduce to the public benefit, and to the usefulness and respectability of the medical practitioner. A sub committee was accordingly appointed to carry these intentions into effect: the gentlemen composing it had an interview with Mr. Brougham, by whom they were received very courteously; and subsequently they conferred with Mr. Hume, who entered with them into a patient and comprehensive investigation, both of the existing Act and of the Bill for its amendment.

" The enlarged view which this distinguished legislator took of the subject, tracing its various bearings as relating both to the community and the profession, fully prepared them to expect from him that distinguished zeal and talent which he has since evinced in his place in Parliament, and which entitle him to the warmest thanks, not only of this Association, but also of every member of the medical community. The Act, however, possesses so many inherent imperfections, that, notwithstanding the proposed amendments, (which, however, will be found to be important meliorations,) it is to be feared it will still prove very inadequate to the more enlightened purposes originally contemplated by this Association.

" The rapid progress in knowledge,—the increasing attention of the legislature to the wants of the community,—and, above all, the intended establishment of a metropolitan university, afford a just expectation that the period is not far distant when the ardent hopes of the Association, as subservient to the best interests of mankind, will be realised.

" Published by order of the general meeting,

" JOHN POWELL, Secretary."

---

### MONTHLY CATALOGUE OF MEDICAL BOOKS.

### METEOROLOGICAL JOURNAL,

By Messrs. WILLIAM HARRIS and Co. 50, Holborn, London.

*From June 20 to July 19, 1825.*

| | MOON. | Rain gauge | THERM. | | | BAROM. | | DE LUC'S HYG. | | WIND. | | ATMOSPHERIC VARIATIONS. | | |
|---|---|---|---|---|---|---|---|---|---|---|---|---|---|---|
| | | | 9 A.M. | MAX. | MIN. | 9 A.M. | 10P.M. | 9 A.M. | 10P.M. | 9 A.M. | 10P.M. | 9 A.M. | 2 P.M. | 10P.M. |
| June | | | | | | | | | | | | | | |
| 20 | | | 60 | 67 | 44 | 29.58 | 29.62 | 66 | 70 | N | NE | Cloud. | Rain | Cloud. |
| 21 | | .22 | 57 | 58 | 45 | 29.70 | 29.79 | 61 | 70 | NW | E | Fine | Fine | Fine |
| 22 | | | 55 | 65 | 53 | 29.90 | 29.90 | 62 | 61 | N | NNW | | | |
| 23 | ☽ | | 58 | 67 | 53 | 29 93 | 29.89 | 62 | 55 | NNW | SW | Cloud. | | Rain |
| 24 | | | 64 | 71 | 56 | 29.82 | 29.75 | 57 | 60 | SW | SW | Fine | | Cloud. |
| 25 | | | 66 | 75 | 53 | 29.63 | 29.54 | 56 | 68 | S | WSW | | Rain | Fine |
| 26 | | .20 | 66 | 71 | 49 | 29.60 | 29.62 | 62 | 67 | W | WSW | | Fine | |
| 27 | | | 62 | 68 | 52 | 29.63 | 29.65 | 62 | 68 | W | WSW | | | |
| 28 | | | 58 | 64 | 52 | 29.60 | 29.50 | 72 | 78 | SSW | WSW | Sho'ry | Rain | Sho'ry |
| 29 | | .28 | 58 | 67 | 59 | 29.53 | 29.52 | 72 | 76 | NW | W | Fine | | |
| 30 | ○ | | 62 | 68 | 55 | 29.52 | 29.52 | 70 | 76 | WNW | WSW | | | Fine |
| July | | | | | | | | | | | | | | |
| 1 | | | 60 | 67 | 50 | 29.64 | 29.87 | 67 | 72 | NW | NNE | | Cloud. | Cloud. |
| 2 | | | 58 | 68 | 52 | 30.05 | 30.10 | 62 | 72 | ENE | NW | | Fine | Fine |
| 3 | | | 63 | 70 | 60 | 30.12 | 30.05 | 62 | 58 | W | NW | | | |
| 4 | | | 66 | 72 | 60 | 30.08 | 30.13 | 67 | 62 | NW | N | | | |
| 5 | | | 65 | 68 | 55 | 30.17 | 30.17 | 64 | 60 | N | N | | | |
| 6 | | | 60 | 65 | 55 | 30.05 | 30.00 | 60 | 66 | NE | E | Cloud. | Sho'ry | |
| 7 | ☾ | .03 | 56 | 66 | 55 | 29.98 | 29.94 | 69 | 75 | NE | NE | | Cloud. | Sho'ry |
| 8 | | .01 | 57 | 60 | 52 | 29.94 | 29.96 | 80 | 80 | NE | N | | | Cloud. |
| 9 | | | 55 | 66 | 55 | 29.95 | 29.92 | 83 | 70 | NNE | NE | | Fine | Fine |
| 10 | | | 62 | 68 | 57 | 29.90 | 29.90 | 62 | 70 | N | WSW | | | |
| 11 | | | 65 | 74 | 57 | 29.90 | 29.90 | 65 | 65 | WSW | WSW | Fine | | |
| 12 | | | 69 | 77 | 67 | 29.91 | 29.93 | 62 | 55 | W | W | | | |
| 13 | | | 71 | 79 | 61 | 30.00 | 30.04 | 57 | 63 | W | WSW | | | Cloud. |
| 14 | | | 69 | 79 | 63 | 30.07 | 29.99 | 73 | 60 | SW | SSW | | | Fine |
| 15 | ● | | 83 | 86 | 67 | 29.91 | 29.94 | 52 | 63 | S | WSW | | | |
| 16 | | | 77 | 85 | 64 | 30.06 | 30.12 | 60 | 58 | N | ESE | | | |
| 17 | | | 76 | 82 | 65 | 30.17 | 30.12 | 59 | 55 | ESE | ESE | | | |
| 18 | | | 76 | 87 | 67 | 30.09 | 30.06 | 65 | 62 | ESE | E | | | |
| 19 | | | 76 | 88 | 64 | 30.10 | 30.18 | 66 | 51 | E | SE | | | |

The quantity of rain fallen in the month of June, was 99.100ths of an inch.

