# Errata in Our Last Number

**Published:** 1825-09

**Authors:** 


Errata *in our last Number.*

In the notice of the *Carob Tree* :—Instead of "arbor quæ *fuit*," read arbor quæ *fert* ;" for "*fabulis*," read "*fabis*;" for "impedes *indigestion*," read "impedes *digestion*."

